# Frequent attenders in the German healthcare system: determinants of high utilization of primary care services. Results from the cross-sectional German health interview and examination survey for adults (DEGS)

**DOI:** 10.1186/s12875-020-1082-9

**Published:** 2020-01-13

**Authors:** Melanie Luppa, Jan Giersdorf, Steffi Riedel-Heller, Franziska Prütz, Alexander Rommel

**Affiliations:** 1 Institute of Social Medicine, Occupational Health and Public Health, University of Leipzig, Leipzig, Germany; 2 Department of Epidemiology and Health Monitoring, Robert Koch Institute, Berlin, Germany; 3 Department of General Practice, Medical Faculty, University of Leipzig, Leipzig, Germany

**Keywords:** Primary care, General practitioner, Family practice, Frequent attendance, Health services, Needs and demand, Social determinants of health, Comorbidity, Mental disorders, Health surveys

## Abstract

**Background:**

In Germany, patients are consulting general practitioners increasingly frequently, resulting in a high burden on the healthcare system. This study aimed to identify factors associated with frequent primary care attendance in the German healthcare system.

**Methods:**

The German Health Interview and Examination Survey for Adults (DEGS) is part of Germany’s national health monitoring, and includes a large representative sample of the German population aged 18–79 years. We defined the 10% of participants with the highest number of general practitioner contacts in the preceding 12 months as frequent attenders of primary care services. Binary logistic regression models with average marginal effects were used to identify potential determinants for frequent use of primary care services.

**Results:**

The sample comprised 7956 participants. Significant effects on frequent use of primary care were observed for low socioeconomic status, stressful life events, factors related to medical need for care such as medically diagnosed chronic conditions and for subjective health. In the full model, the number of non-communicable diseases and subjective health status had the strongest effect on frequent primary care use. We found an interaction effect suggesting that the association between subjective health status and frequent attendance vanishes with a higher number of non-communicable diseases.

**Conclusions:**

We observed strong associations between frequent primary care attendance and medical need for care as well as subjective health-related factors. These findings suggest that better coordination of care may be a preferred method to manage health services utilization and to avoid redundant examinations and uncoordinated clinical pathways. Further research is needed to clarify moderating and mediating factors contributing to high utilization of primary care services.

## Background

Increasing health service use is a common issue in European healthcare systems [1], particularly the increasing use of primary care. In Germany, patients are consulting general practitioners (GPs) with increasing frequency [2]. However, the highest workload for GPs is often associated with a small group of chronically ill patients with a high number of contacts; this group is termed high users or frequent attenders [3–7].

Frequent attenders are patients who attend GPs on a regular basis and exceed a certain number of visits within a given period [8–10]. They consume large amounts of primary care resources, resulting in high cost to the healthcare system [11–13]. Currently, there is no widely accepted definition of frequent attendance [5], although such a definition may impact on the results of studies investigating this issue. Many of the previous studies used proportional approaches, and considered differing quantiles of patients with the highest number of physician contacts as frequent attenders [5].

Several studies have analysed frequent attenders and related factors. Age and female sex are commonly reported as determinants of frequent attendance [3, 5, 14–17]. Many studies have reported other strongly associated factors, such as severe or chronic physical disease [16, 18–20] and mental health problems [16, 18, 20]. In particular, patients with a high number of chronic diagnoses showed a 50% increased risk for being classified as frequent attenders [21]. Frequent attendance among patients with mental health problems may result from a more frequent presentation of unspecific medical complaints, high stress burden and increased anxiety or somatisation levels, which lead to increased medical treatments and prescriptions [22–27]. Sociodemographic factors are also associated with frequent attendance, although previous studies reported inconsistent findings [5, 28]. Other contributing factors reported in some studies included psychosocial stressors such as a distorted family life, stressful life events or other social problems (e.g. low social support or loneliness) [23, 29]. In contrast, findings regarding associations between frequent attendance and unemployment, early retirement and sick leave are relatively consistent across studies [4, 5, 14, 15, 30].

The present study aimed to identify sociodemographic, psychosocial and health-related factors associated with frequent primary care attendance in the German healthcare system, using a large representative sample of the German population aged 18–79 years. The German Health Interview and Examination Survey for Adults (DEGS) is a comprehensive health interview and examination survey [21, 31, 32]. It allows for analysis of frequent attendance related to a broad spectrum of medically diagnosed diseases, psychometric tests, sociodemographic and psychosocial determinants and subjective factors such as self-rated health. It was hypothesized that sociodemographic and psychosocial factors remain associated with the frequent use of GP services, regardless of the control of health-related factors. Secondly, an association between subjective health and frequent GP use independent of the presence of medically diagnosed diseases was assumed. As a secondary objective of the study, interaction analyses were conducted to test the assumption that factors such as social support or partnership can have an effect dependent on age and sex. In a similar way, it was tested whether the effect of self-perceived health on the frequent use of GPs depends on the number of medically diagnosed diseases.

## Methods

### Data collection

The German Health Interview and Examination Survey for Adults (DEGS) is part of the health monitoring conducted by the Robert Koch Institute. The design and methodical details of the DEGS study have been described elsewhere [33, 34]. The DEGS study was conducted from 2008 to 2011 and included interviews, examinations and tests which were carried out in temporary study centres [35]. The main part of the information was collected via self-administered questionnaires. The target population was residents of Germany aged 18–79 years. The DEGS study used a mixed design that permits both cross-sectional and longitudinal analyses. The sample included former participants from the German National Health Interview and Examination Survey 1998 (GNHIES98) that were interviewed and examined for the second time, along with a newly drawn random sample. Both studies followed a cluster sampling approach drawing participants from local population registries for equally distributed sample points. In total, 8151 persons participated in the DEGS study; 4192 first-time participants (response rate 42%) and 3959 GNHIES98 participants (response rate 62%) [34]. The net sample allows representative cross-sectional and time trend analyses for people aged 18–79 years, excluding 165 revisiting GNHIES98 participants who were over age 79 years. Pregnant women (*n* = 31) were also excluded from the sample because they have many primary care visits over a short time. Therefore, the total sample for the present analyses comprised 7956 participants.

### Variables

#### Outcome variable

So far, there is no standardized definition of how the group of FAs should be distinguished from the “normal” utilizers [5, 28]. On the one hand, an absolute cut-off value such as 6 GP contacts per year can be determined. On the other hand, proportional boundaries are chosen, such as the 25% or 10% of respondents with the most GP contacts. The latter approach has the advantage of better comparability across studies and countries [28]. On the contrary, the absolute number of physician contacts depends on legal regulations and care settings. Absolute thresholds therefore can only be justified for the healthcare settings under study. As there is no well-justified absolute threshold for the definition of FAs in primary care in Germany the present study relied on a proportional approach.

The number of GP contacts in the 12 months before the DEGS interview was derived from participants’ answers to the question: ‘Please tell us how often you used outpatient services for the following specialties in the past 12 months?’ For the present study, only contacts with primary care specialists (GPs) were considered. We defined frequent attenders as the 10% of participants with the highest number of GP contacts in the 12 months before the interview. The data set was first divided into six age- (18–39 years, 40–59 years, 60 + years) and sex-specific strata. In each of these subsets, the 10% of the population with the highest number of contacts to GPs in the last year before the interview were identified. This information was converted into a dichotomous variable (frequent attendance of primary care yes/no). Finally, these subsets were merged again in order to generate a uniform variable of use frequency across all age- and sex-specific strata. The reason for this approach was that women and older people more frequently use outpatient services [36]. Therefore, without stratification, young and male frequent primary care users would be underrepresented in the frequent attenders group. Decisions regarding the 90th percentile and stratification were based on recommendations suggesting that this definition offered the best discrimination between ‘normal’ users and frequent attenders [4, 37, 38].

#### Sociodemographic determinants

Socioeconomic status (SES) was determined using an index that included information on education and vocational training, professional status and net household income (weighted by household needs), which allowed classification into low, middle or high SES groups [39]. A migrant background was assumed if the respondent or one of their parents was born abroad [40, 41]. Finally, people living in marriage or consensual unions were distinguished from those not currently in a relationship.

#### Psychosocial stress variables

Social support (low vs. moderate/high), long-term unemployment (yes/no), at least one stressful life event (yes/no) and early retirement (yes/no) were included as psychosocial stress factors. Social support was measured by dividing the Oslo-3 Social Support Scale into two categories (low and moderate/high) [42]. Long-term unemployment was defined as more than 12 months of unemployment during the last 5 years. Participants were asked whether they had experienced at least one of 10 stressful life events during the last 12 months: (i) death of their spouse, (ii) separation or divorce, (iii) death of a related person, (iv) own serious illness, (v) own serious accidental injury, (vi) transition to retirement, (vii) serious illness of a related person, (viii) wartime experience, (ix) experience related to the German wall or the German Democratic Republic political system or (x) others.

#### Medical need for care

Current depressive symptoms and the number of prevalent non-communicable diseases (NCDs) were used as indicators of medical need for care. Depressive symptoms were measured by the 8-item depression module of the Patient Health Questionnaire (PHQ-8) [43]. The PHQ-8 measures depressive symptoms in the last 2 weeks. The cut-off score for depressive symptoms was set at 10 [44]. The PHQ-8 is a reliable and valid screening instrument that has been frequently used in clinical contexts and population-based studies [45, 46].

Self-reported medical diagnoses were collected and validated during an additional physician-assisted face-to-face interview. Information on diagnoses were aggregated to a summary score showing the number of prevalent NCDs. The measure included 12-month prevalence of depression, anxiety disorders, burn-out, eating disorders, bronchial asthma, allergic diseases, inflammatory bowel disease, diabetes, lipometabolic disorder, epilepsy, hepatitis, heart failure, hypertension, uric acid increase, gout, migraine, thyroid disease, gastric/duodenal ulcers and diseases not otherwise expressly mentioned (‘further diseases’). Chronic diseases (degenerative joint disease including osteoarthritis, osteoporosis, cancer, coronary heart disease including myocardial infarction, stroke, cirrhosis, chronic renal insufficiency, Parkinson’s disease and prostatic hyperplasia) were included in the summary score as life-time prevalence.

#### Subjective health status

Subjective health status was measured using three indicators. General health was assessed based on indicators from the European Community Health Indicators Monitoring [43, 47]. Self-rated health was explored by the question: ‘How is your health in general?’ Responses were recorded as a dichotomous variable (very good/good vs. moderate/worse). Global activity limitations were assessed by the question: ‘For at least the past 6 months, to what extent have you been limited in activities people usually do because of a health problem?’ Respondents who reported being either ‘limited’ or ‘strongly limited’ in their daily activities were aggregated into one category to give the proportion of individuals with limitations. In addition, the statement ‘I seem to get sick a little easier than others’ (answered with ‘yes’ or ‘no’) was used as indicator of self-rated vulnerability, on the assumption that perceived threat from disease affected health service use [48].

### Statistical analyses

All analyses were conducted with Stata 15.1 (Stata Corp., College Station, TX, USA, 2017) using survey procedures for complex samples. This allowed us to adequately account for the clustering of participants in sample points and consider weighting in calculating confidence intervals and *p*-values. Weighting factors were used to correct deviations in the sample from the population structure regarding age, sex, region, nationality, community type, education level and the re-participation probability of GNHIES98 participants in order to enable representative statements for the German population (reference date 31.12.2010). Multivariate binary logistic regression was used to evaluate associations between various determinants and frequent attendance of primary care services. Average marginal effects (AME) were calculated to overcome the problem of unobserved heterogeneity that impedes direct comparisons of odds ratios between different models [49, 50]. AME indicate the increase in percent of the probability of an event (dependent variable) if the independent variable changes by one unit [51]. The change in AME between different models can be directly interpreted [49, 50].

The analyses followed a blockwise modelling approach. Model 1 assessed the association between sociodemographic factors and frequent attendance. Model 2 explored the extent to which psychosocial stressors determined frequent attendance if sociodemographic factors were controlled. Model 3 quantified the impact of medical need for care and revealed whether the effects of sociodemographic factors and psychosocial stressors persisted independent of medical need. Finally, Model 4 assessed the contribution of subjective health-related factors to the explanation of frequent attendance. This blockwise modelling allowed us to quantify the extent to which the effect of single factors decreased by adding further dimensions.

To identify age- and sex-specific determinants of frequent attendance, Model 4 was also calculated separately for women and men, and for younger (18–64 years) and older (65+ years) respondents (results not shown). For effects that were significant for only one sex or age group, interactions between sex/age and the respective factor were tested for statistical significance. Moreover, we tested if there were significant interactions between subjective health and medical need factors. To better illustrate the findings, model-based predictive probabilities for frequent attendance conditional on certain combinations of determinants were calculated and visualised (adjusted predictions at representative values; APM). The APM provides the average prevalence of the outcome when certain determinants are held constant [51].

## Results

The sample comprised 7956 participants; 49.9% were male and 50.1% were female. Age was almost normally-distributed between 18 and 79 years, with the group aged 40–54 years being the largest and accounting for 31.3% of the overall sample. Further sample characteristics are shown in Table 1.

**Table 1:** Sample characteristics

		n	% weighted	95% CI	
Sex	male	3789	49.9	48.4	51.3
	female	4167	50.1	48.7	51.6
	missing	0			
Age	18–24	625	10.5	9.8	11.2
	25–39	1432	22.8	21.6	24.0
	40–54	2398	31.3	30.4	32.3
	55–69	2268	22.6	21.7	23.6
	70+	1233	12.8	12.1	13.5
	missing	0			
Socioeconomic status	low	1234	19.7	18.3	21.3
	medium	4728	60.3	58.8	61.8
	high	1904	20.0	18.5	21.5
	missing	90			
Migrant background	no	6571	80.2	78.1	82.2
	yes	1100	19.8	17.8	21.9
	missing	285			
Marriage/consensual union	yes	6283	79.6	78.3	80.9
	no	1480	20.4	19.1	21.7
	missing	193			

Source: DEGS (*n* = 7956)

The multivariate analysis (n = 6730 without cases with missing data) of the association between sociodemographic factors and frequent primary care attendance showed a significant effect only for SES. Compared with the high SES group, participants in the middle SES group, had a 3.5% increased probability of being a frequent attender; if they were in the low SES group, the probability increased by 9.0% (Table 2, Model 1). Comparing Model 1 with Model 4 revealed that a considerable part of these effects were explained by other factors such as psychosocial stress, medical need for care and subjective health status. Overall, the effect size for SES was reduced by 51% in individuals in the low SES group, and by 43% in those in the middle SES group. In both groups, more than half of the reduction in effect was associated with the inclusion of subjective health status-related factors in Model 4.

**Table 2:** Social and health related determinants for frequent primary care attendance: Results of binary logistic regression analyses (average marginal effects)

	Indicator	Category	Model 1			Model 2			Model 3			Model 4		
	Variable		AME	95%	CI	AME	95%	CI	AME	95%	CI	AME	95%	CI
Sociodemographics	SES [Ref high]	low	**0.090**	0.058	0.121	**0.075**	0.046	0.105	**0.069**	0.042	0.097	**0.044**	0.017	0.070
		medium	**0.035**	0.017	0.052	**0.032**	0.014	0.049	**0.028**	0.010	0.046	**0.020**	0.001	0.038
	Migrant background [Ref yes]	no	0.012	−0.011	0.036	0.013	− 0.010	0.035	0.015	−0.007	0.038	0.015	−0.007	0.038
	Marriage/consensual union [Ref yes]	no	0.007	−0.014	0.029	−0.004	−0.024	0.016	−0.005	− 0.025	0.014	− 0.003	−0.023	0.016
Psychosocial stress	Social support [Ref moderate/high]	low				0.007	−0.018	0.033	−0.009	− 0.032	0.015	− 0.016	−0.039	0.007
	Unemployment [Ref no]	yes				0.027	−0.012	0.067	0.013	−0.024	0.049	0.004	−0.031	0.039
	Early retirement [Ref no]	yes				**0.198**	0.110	0.286	**0.099**	0.017	0.181	0.063	−0.011	0.137
	Stressful life event [Ref no]	yes				**0.061**	0.040	0.082	**0.031**	0.011	0.052	**0.023**	0.003	0.042
Medical need	NCDs [continuous]	count							**0.028**	0.023	0.034	**0.019**	0.013	0.025
	Current depressive symptoms [Ref no]	yes							**0.047**	0.006	0.088	0.017	−0.017	0.050
Subjective Health	Self-rated health [Ref (very) good]	moderate or worse										**0.084**	0.056	0.112
	Global activity limitations [Ref no]	yes										0.024	−0.007	0.055
	More likely to get sick [Ref no]	yes										**0.063**	0.031	0.096

Source: DEGS (*n* = 6730)

*AME* average marginal effects, *CI* confidence interval, *SES* socioeconomic status, *NCDs* non-communicable diseases. Boldface indicates *p* < 0.05

Model 2 explored the effects of psychosocial stress factors controlled for sociodemographic factors. Early retirement and stressful life events showed positive significant effects on frequent primary care attendance (Table 2). After inclusion of medical need for care and subjective health status in Models 3 and 4, the effect sizes for stressful life events and early retirement were reduced by 63 and 68%, respectively. In Model 4, early retirement no longer had a significant effect, whereas the probability of frequent primary care attendance was still increased by 2.3% with the presence of stressful life events.

In Model 3, medical need (number of NCDs and current depressive symptoms) was significantly associated with frequent attendance (Table 2). After inclusion of subjective health status factors, current depressive symptoms no longer had a significant effect (Model 4). However, number of NCDs showed a more stable association with frequent attendance. After inclusion of subjective health status in the full model, the probability of being a frequent attender was still increased by 1.9% with each further disease. Overall, in Model 4, the effect size reduction in comparison with Model 3 was 65% for depressive symptoms and 33% for number of NCDs. In the full model (Model 4), subjective health status-related factors showed the strongest effect on frequent primary care attendance. A moderate/worse subjective health status increased the probability of frequent primary care attendance by 8.4%. The perception of getting sick more easily than others increased the probability of frequent attendance by 6.3% (Table 2).

**Fig. 1:**
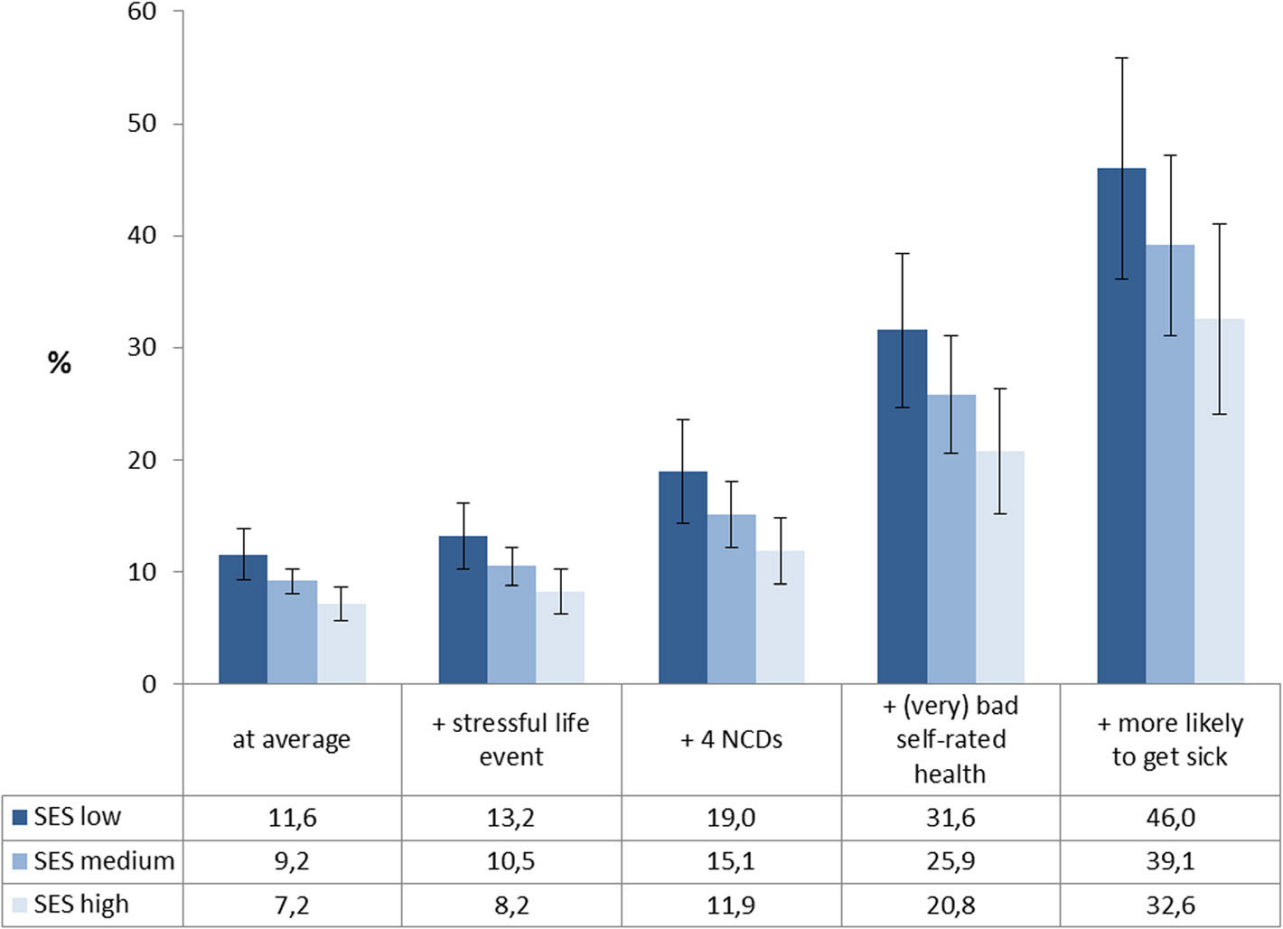
Cumulated effects on the frequent use of primary care by socioeconomic status (SES) (model-based predictions in %). Source: DEGS (*n* = 6730)

Figure 1 shows the cumulated effects for frequent primary care attendance by SES expressed as model-based predicted probabilities based on Model 4. On average, 7.2% of individuals with high SES and 11.6% with low SES were frequent attenders. These percentages increased with each additional risk factor. Inclusion of all significant determinants resulted in 46.0% of frequent attenders in the low SES group and 32.6% in the high SES group.

**Fig. 2:**
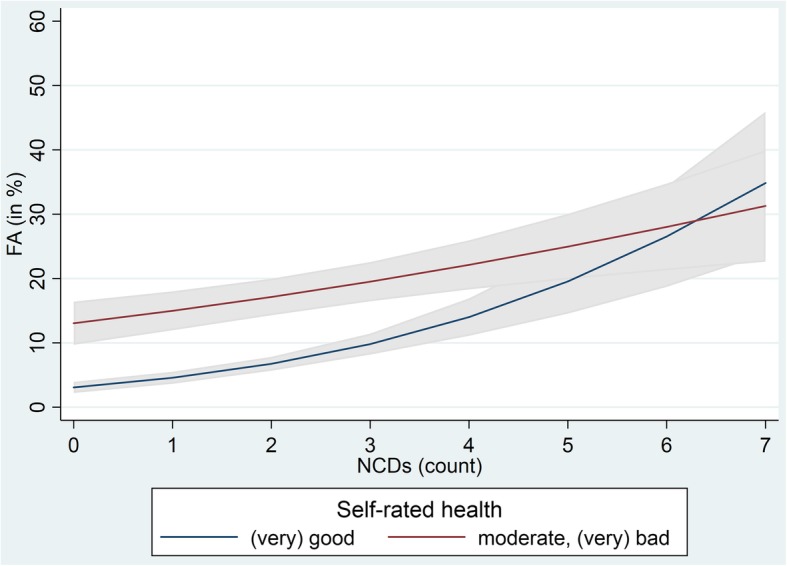
Frequent primary care attenders (FA) by number of non-communicable diseases and self-rated health (model-based predictions in %). Source: DEGS (*n* = 6730)

Analyses of interactions revealed no significant interaction effects between age and other factors on frequent primary care attendance. In contrast, significant interaction effects were found between sex and migrant background, and sex and social support. Only women with a migrant background were less often frequent attenders than women without a migrant background (model-based predictions: 6.7% vs. 10.6%). Only men with low social support were less often frequent attenders than men with moderate or high social support (model-based predictions: 5.7% vs. 9.3%). A significant interaction effect on frequent primary care attendance was also found between number of NCDs and subjective health status (Fig. 2); the lower the number of NCDs, the higher the effect of subjective health status on frequent primary care attendance. In cases of no or few NCDs, frequent attendance was clearly associated with subjective health status. This effect vanishes with number of NCDs. For example, about one-third of the individuals with six NCDs were classified as frequent attenders independent of subjective health status (Fig. 2). Comparable results were found for the interaction of number of NCDs and the perception of getting sick more easily than others (results not shown).

## Discussion

This study aimed to identify factors associated with frequent primary care attendance in the German healthcare system. Based on DEGS data, representative statements can be made about health status, health behaviour and health service use in the German population**.** In the present study, we defined the 10% of patients with the highest number of GP visits in the preceding 12 months as frequent attenders. Previous studies showed that a cut-off of 90% better discriminated frequent attenders and ‘normal’ users than other quantiles (e.g. 75%) and support the 90th percentile as an adequate definition of frequent attendance [37, 38]. It is also recommended to stratify such a definition by age and sex to achieve sufficient specificity and sensitivity of the measurement [4, 37]. According to a recent review, studies with case definitions based on absolute thresholds show rates of FAs between 14 and 33% [28]. It can therefore be considered as a disadvantage of the proportional approach that only part of the phenomenon that causes an increased GP workload is covered [28]. However, there are hardly any reasonable criteria for defining FAs based on absolute thresholds that could be applied to different settings and countries. Thus, the use of proportional criteria makes it possible to standardize research on frequent attendance [4, 38]. Like other proportional definitions that are used for international comparisons (such as the relative definition of income poverty), proportional thresholds better allow for comparisons across studies and countries.

### Morbidity and subjective health status

In general, the present study showed strong associations between frequent primary care attendance and medical need for care. In particular, the number of NCDs was strongly associated with frequent attendance. Similar results were found in systematic reviews conducted by Vedsted and Christensen [5] and Welzel et al. [28]. Both reviews reported positive associations between severity of physical disorders and multimorbidity and frequent attendance. Moreover, current German studies have consistently shown that frequent attenders suffer from chronic conditions, severe illnesses or multimorbidity more often than non-frequent attenders [21, 52, 53]. In particular, chronic conditions such as osteoarthritis, rheumatoid arthritis and other diseases of the musculoskeletal system, respiratory diseases, migraine and back pain were associated with frequent attendance [4, 5]. Van den Bussche et al. (2016) analysed claims data for a German statutory health insurance company and reported 27 chronic conditions in persons aged ≥65 years that doubled the risk for frequent attendance [21].

Subjective health status-related factors also had strong effects on frequent attendance that were independent from medical need for care. In particular, moderate or poor self-rated health status and the perception of getting sick more easily than others increased the risk for frequent attendance by 8 and 6%, respectively., We found only few previous studies that have assessed these factors [32, 52, 54]. These have consistently shown a clear positive association between subjective health status and frequent attendance. This is underpinned by a recent concept analyses that identified low self-rated health and poor quality of life as defining attributes of FAs in primary care. Higher risks for frequent attendance have as well been reported for individuals with mental health problems or psychological distress [5, 21, 55–57], somatisation [25, 26], depressive symptoms [55, 58] and increased anxiety levels [23, 29], which are often strongly associated with a poor self-rated health status [59, 60]. In our study, depressive symptoms (as assessed by the PHQ-8) no longer showed a significant effect after inclusion of subjective health status-related factors. Other studies have also questioned the effect of mental health problems on frequent attendance, suggesting an overestimation of this effect and noting the advice in medical guidelines relating to regular physician visits as a mediating factor [4]. The present analysis highlighted the importance of further research on frequent attendance considering the interplay between somatic conditions, mental health problems and subjective health status-related factors. This was also underlined by the findings of the interaction analyses showing that the association between subjective health-related factors and frequent attendance depend on the degree of medical need (here number of NCDs).

### Sociodemographic variables and psychosocial stress

Compared with health-related variables, sociodemographic and psychosocial factors showed less impact on frequent attendance and are only partially health-independent determinants of frequent GP use. We found significant effects on frequent attendance only for low SES and presence of at least one stressful life event in the preceding 12 months (Models 1–4). The effect of low SES on frequent primary care attendance, even when health-related variables and morbidity were controlled, can be consistently seen in previous studies [61, 62]. Correspondingly, specialised care is more often observed in higher SES groups [61]. Moreover, the present study provided no evidence to support the hypothesis that frequent attendance was associated with loneliness and old age. Although we did not directly measure loneliness, social support as a proxy showed no impact on frequent attendance. Finally, the interaction analyses did not show any evidence for age-specific effects and rather weak evidence for sex-specific effects.

### Strengths and weaknesses

The DEGS study was designed to provide representative statements of the health status, health behaviour and use of medical care for the German population aged 18–79 years, and allows analyses of time trends in population health. Bias resulting from selective participation of healthier persons, which is a known concern for population-based surveys, might have led to an underestimation of the overall prevalence of chronic diseases compared with results from claims data. In addition, persons unable to provide written consent and those with significant language barriers were excluded from participation in the DEGS study. When interpreting the results it should be borne in mind that the data on GP contacts are based on self-reported data that may be prone to recall bias [63, 64]. However, there is some evidence showing that there is considerable correspondence between self-reported data and accounting data when it comes to the utilization prevalence [65]. Nevertheless, it should be considered that self-reported GP contacts are rather a rough approximation to the actual level of GP-patient contacts and that a proportional definition of frequency attenders is not an exact definition of this group. To ensure that estimates derived from the DEGS study are representative at the national level, weighting factors were applied. Furthermore, the DEGS has a cross-sectional design and identified associations should not be mistaken for causal relationships.

## Conclusions

According to the findings of the present study, frequent use of GPs is mainly associated with health-related factors, which can be influenced to a certain extent by the health care system. Thus, solutions should be sought that focus on improvement of health care rather than economic disincentives. Approaches such as co-payments aimed at lowering the use of services have not proven to lead to the desired outcomes [66]. In the case of chronic diseases, for example, the quality of care can be a critical factor. Future research should focus on finding out which improvements would be promising in order to diminish frequent attendance (e.g. coordination of care). Furthermore, the influence of stressful life events indicates that it is important for people in difficult life situations to have a specialized psychosocial care. Moreover, the positive association between a low self-rated health and frequent attendance suggests that in primary care a focus on the preservation of the quality of life of persons with NCDs may be another promising approach to reduce frequent attendance. A useful objective of further studies could therefore be to investigate whether frequent attendance in chronically ill persons is lower if a certain degree of functionality and quality of life is maintained for as long as possible. Finally, it should be noted that frequent primary care attendance occurs in a multicomplex context. It is based on underlying somatic diseases as well as psychological complaints and the characteristics of the healthcare system. The present study only partially addressed these interdependencies. Therefore, further research is needed to clarify moderating and mediating factors contributing to high use of primary care services. In particular, studies with a longitudinal design may help to better identify causes of frequent attendance.

## Data Availability

The ‘Health Monitoring’ Research Data Centre at the Robert Koch Institute (RKI) is accredited by the German Data Forum according to uniform and transparent standards (http://www.ratswd.de/en/data-infrastructure/rdc). The DEGS data set is freely accessible on application to interested scientists as de facto anonymized data for scientific secondary analysis. More detailed information on access, application forms and guidelines can be obtained from datennutzung@rki.de.

## Abbreviations

AME: Average marginal effects
APM: Adjusted predictions at representative values
DEGS: German Health Interview and Examination Survey for Adults
GNHIES98: German National Health Interview and Examination Survey 1998
GP: General practitioner
NCDs: Non-communicable diseases
PHQ 8: Patient Health Questionnaire - 8
SES: Socio-economic status
